# Current distribution of selected canine vector‐borne diseases in domestic dogs from Barranquilla and Puerto Colombia, Atlántico, Colombia

**DOI:** 10.1002/vms3.673

**Published:** 2021-12-03

**Authors:** Óscar Otalora, Guillermo Couto, Julio Benavides, Carlos Mucha, Rodrigo Morchón

**Affiliations:** ^1^ Clinica Veterinaria Vida Animal del Caribe Barranquilla Colombia; ^2^ Couto Veterinary Consultants Hilliard Ohio USA; ^3^ Departamento Administrativo Distrital de Salud (DADIS) Cartagena Colombia; ^4^ Práctica Privada en Cardiología Buenos Aires Argentina; ^5^ Zoonotic diseases and One Health Group Laboratory of Parasitology Faculty of Pharmacy University of Salamanca Salamanca Spain

**Keywords:** *Anaplasma* spp, *Borrelia burgdorferi*, Colombia, *Dirofilaria immitis*, *Ehrlichia* spp

## Abstract

**Background:**

Climate change, the increase of travel with infected animals from endemic areas, the introduction of new vectors in these areas and environmental changes caused by human activity, among other factors, have contributed to the establishment and increase of canine vector‐borne diseases (CVBDs), several of which are zoonotic and pose a risk to the human population. In Colombia, there are very few studies that address the prevalence of these diseases. The objective of this study was to update the prevalence of cardiopulmonary dirofilariosis, anaplasmosis, ehrlichiosis and Lyme borreliosis in dogs in Barranquilla and Puerto Colombia, areas of northern Colombia.

**Materials and methods:**

The present study included 354 dogs presented to veterinary clinics for routine health examination and foundations for stray dogs between November 2016 and July 2018.

**Results:**

The percentage of dogs positive for *Ehrlichia* spp. was 61.86%, followed by 22.03% for *Anaplasma* spp., 11.30% positive for *Dirofilaria immitis* antigens and 0.56% positive for *Borrelia burgdorferi*. In addition, several dogs positive for antibodies against two or more infectious diseases were found. Higher seroprevalences were documented in outdoor dogs compared to indoor‐housed dogs.

**Conclusion:**

These results suggest that veterinarians should routinely implement prophylactic programmes for these CVBDs, particularly for dogs that reside outdoors.

## Graphical abstract

The objective of this current study was to update the prevalence of cardiopulmonary dirofilariosis and seroprevalence of anaplasmosis, ehrlichiosis and Lyme borreliosis in dogs in Barranquilla and Puerto Colombia, northern Colombia. The prevalence or seroprevalence of four canine vector‐borne diseases has been updated in Atlántico, Colombia, being the highest infection by *Ehrlichia* spp., followed by *Anaplasma* spp., *Dirofilaria immitis* and *Borrelia burgdorferi*, with a high percentage of co‐infections. Most of the infected dogs lived outside houses and were of a high age. Taking into account the location of the infected animals, conditions of humidity and temperature and the presence of vectors, control measures should be taken to minimize the incidence of these diseases.

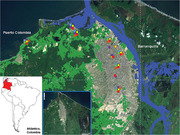

## 1 INTRODUCTION

Canine vector‐borne diseases (CVBDs) pose a high risk to animal (wild/domestic) reservoirs, and due to their zoonotic potential, they also pose a risk to human public health. The prevalence or seroprevalence of CVBDs is influenced by anthropogenic, socio‐economic and demographic factors, increased international trade and the transport of infected pets from endemic areas, environmental changes related to human activities and climate change, as well as the presence of vectors in a given location (Maggi & Krämer, 2019; Morchón et al., 2012; Montoya‐Alonso et al., 2020).

Heartworm disease, caused by *Dirofilaria immitis*, is a cosmopolitan zoonotic disease transmitted by culicid mosquitoes. Domestic dogs are the definitive host, although the disease also affects other carnivores, both domestic and wild. It is a chronic and potentially lethal disease which affects the vascular endothelium and lung parenchyma, while also potentially affecting the right heart chambers, which could lead to congestive heart failure in the infected host (Morchón et al., 2012; Simón et al., 2012).

Canine granulocytic anaplasmosis (CGA), canine cyclic thrombocytopenia (CCT) and canine monocytic ehrlichiosis (CME) are caused by *Anaplasma phagocytophilum*, *Anaplasma platys* and *Ehrlichia* spp., respectively, which mainly affect tropical and subtropical regions (Chomel, 2011). *Borrelia burgdorferi* is the causative agent of Lyme borreliosis, which affects both dogs and humans. The aforementioned parasites are intracellular Gram‐negative bacteria transmitted by different species of ticks (Dumler, 2001).

The majority of dogs with CGA are mainly characterized by having non‐specific signs of fever, anorexia, lethargy, joint pain, vomiting, diarrhoea, anaemia, etc. Dogs with CTT often lack clinical signs despite clinicopathologic evidence of thrombocytopenia, and further are not expected to be anaemic unless thrombocytopenia were to lead to clinically relevant spontaneous haemorrhage, which is unlikely. Dogs with CME can display a variety of clinical signs during the acute phase such as fever, lethargy, poor appetite, chronic eye inflammation, abnormal bruising and bleeding, among other clinical signs (Beaufils et al., 2002). Finally, *Ehrlichia ewingii* infection causes polyarthritis and, in the dog, Lyme borreliosis can cause chronic lameness and joint pain, and if not treated, has the potential to cause glomerulonephritis and cardiac disease. The clinical diagnosis of borreliosis in dogs is complicated since it shares clinical signs with other CVBDs (Maggi & Krämer, 2019).

There are few studies that address the status of these canine diseases in Colombia. *D. immitis* has been reported in different regions of Colombia with prevalence rates ranging from 0.91% to 24% (Dantas‐Torres, Otranto, 2013; Esteban‐Mendoza et al., 2020; N. Labarthe & Guerrero, 2005; L. Labarthe et al., 2018; McCown et al., 2014). Pesapane et al. (2019) reported the seroprevalence of *Ehrlichia canis* (15.3%), *A. platys* (20.2%) and both (6.5%) in Santa Marta and Ciénaga, two regions localized in northern Colombia, near Barranquilla. In Barranquilla, there are studies that indicate a seroprevalence of 32.7%–83% for *E. canis*, 2%–40% for *A. phagocytophilum* and a prevalence of 2% for *D. immitis* and even, with some cases presenting antibodies against both infections (*E. canis* and *A. phagocytophilum*) at the same time (L. Labarthe et al., 2018; McCown et al., 2014). In Villavicencio and Bucaramanga (central and northern Colombia, respectively), the seroprevalence of anaplasmosis was 1.1% (Vargas‐Hernández et al., 2016), while in Córdoba (northern Colombia), the seroprevalence of Lyme disease was 20% (Miranda et al., 2009).

Due to the fact that there are few studies that address these diseases and that their prevalence or seroprevalence is very disparate, the objective of this current study was to update the prevalence of cardiopulmonary dirofilariosis and seroprevalence of anaplasmosis, ehrlichiosis and Lyme borreliosis in dogs in Barranquilla and Puerto Colombia, northern Colombia.

## 2 MATERIALS AND METHODS

### 2.1 Characteristics of Barranquilla and Puerto Colombia

**FIGURE 1:**
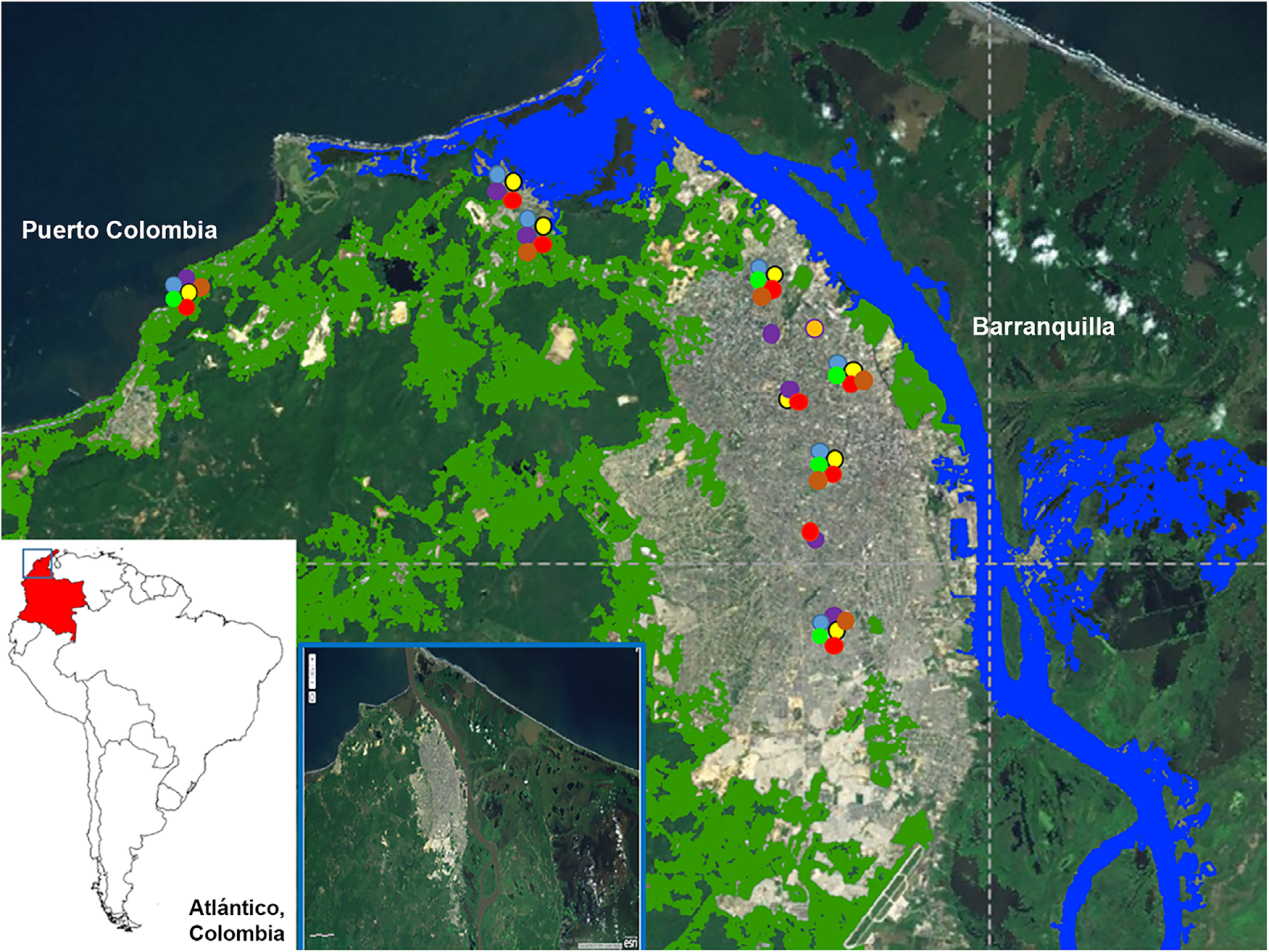
Geographical location of Barranquilla and Puerto Colombia, Atlántico, Colombia. Places: 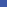 water areas and riverbeds with vegetation and 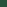 parks and forest. Positive dogs: 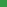 *Dirofilaria immitis*, 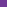 *Ehrlichia* spp., 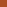 *Anaplasma* spp., 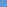 *Dirofilaria immitis* + *Ehrlichia* spp. + *Anaplasma* spp., 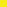 *Dirofilaria immitis* + *Ehrlichia* spp., 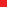 *Ehrlichia* spp. + *Anaplasma* spp. and 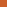 *Borrelia burgdorferi*

Barranquilla and Puerto Colombia are located in Atlántico, northern Colombia, at sea level, with the highest altitude (120 m) at coordinates 10° 59′ 16″ N/ 74° 47′ 20″ O (Figure 1). These regions occupy an area of 154 km^2^ and has 1,228,300 inhabitants, making it the fourth most populated city in the country, with high levels of poverty. During the course of the year, the temperature generally varies from 24 to 32°C and rarely falls below 23°C or rises above 34°C. The rainy season lasts 8.4 months, from April to December. The humidity level remains almost constant at 100%. These regions are surrounded by extensive cultivated areas and within the urban area there are green spaces, rivers with vegetation and highly polluted streams and swamps.

### 2.2 Dog samples

This study included 354 dogs presented to one veterinary clinic and two foundations for stray dogs in Barranquilla between November 2016 and July 2018. In all cases, the same veterinarian conducted a physical examination and recorded any clinical signs. The inclusion criteria were dogs over 6 months of age that had not travelled outside the area of interest of the study in the last 6 months, not receiving regular chemoprophylaxis or vector repellents for the studied vector‐borne diseases and with the owner's informed consent to participate in this study. Dogs were randomly selected by convenience sampling having the permission of the owner or staff of the shelter from which the dog samples were obtained. From each dog, the following data were collected: age, gender, breed, and zip code of their home address (Table 1).

**TABLE 1:** Prevalence and seroprevalences using the 4DX SNAPW Test (IDEXX Laboratories, Inc., Westbrook, Maine, USA) for 354 dogs analyzed for *Dirofilaria immitis* antigens and *Ehrlichia* spp., *Anaplasma* spp. and *Borrelia burgdorferi* antibodies and in Barranquilla and Puerto Colombia, Atlántico, Colombia, according to different categories. (%) Percentage of dogs; (+) positive dogs; (*n*) number of dogs sampled

	*D. immitis* % (+/*n*)	*Ehrlichia* spp. % (+/*n*)	*Anaplasma* spp. % (+/*n*)	*B. burgdorferi* % (+/*n*)
Age				
<1	7.25 (5/69)	43.48 (30/69)	18.84 (13/69)	0 (0/69)
1–2.9	11.29 (7/62)	66.13 (41/62)	11.29 (7/62)	0 (0/62)
3–4.5	15.38 (14/91)	70.33 (64/91)	30.77 (28/91)	0 (0/91)
4.6–7.4	9.23 (6/65)	75.38 (49/65)	30.77 (20/65)	3.08 (2/65)
>7.5	11.94 (8/67)	52.24 (35/67)	14.93 (10/67)	0 (0/67)
Sex				
Male	10.06 (16/159)	56.60 (90/159)	18.24 (29/159)	1.12 (2/159)
Female	12.31 (24/195)	66.15 (129/195)	25.13 (49/195)	0 (0/195)
Habitat				
Indoor	3.33 (1/30)	40 (12/30)	3.33 (1/30)	0 (0/30)
Outdoor	12.04 (39/324)	63.89 (207/324)	23.77 (77/324)	0.62 (2/324)
Breed				
Beagle	11.76 (2/17)	23.53 (4/17)	5.88 (1/17)	5.88 (1/17)
Boxer	0 (0/1)	0 (0/1)	0 (0/1)	0 (0/1)
English Bulldog	20 (1/5)	60 (3/5)	40 (2/5)	0 (0/5)
French Bulldog	0 (0/2)	0 (0/2)	0 (0/2)	0 (0/2)
Cocker Spaniel	0 (0/4)	50 (2/4)	25 (1/4)	0 (0/4)
Dalmatian	50 (1/2)	50 (1/2)	0 (0/2)	0 (0/2)
Dogue de Bordeaux	0 (0/1)	100 (1/1)	0 (0/1)	0 (0/1)
French Poodle	7.14 (2/28)	53.57 (15/28)	14.29 (4/28)	0 (0/28)
Greyhound	0 (0/1)	100 (1/1)	0 (0/1)	0 (0/1)
Golden	0 (0/3)	66.67 (2/3)	66.67 (2/3)	0 (0/3)
Jack Rusell	0 (0/1)	0 (0/1)	0 (0/1)	0 (0/1)
Labrador	11.11 (1/9)	66.67 (6/9)	0 (0/9)	0 (0/9)
German shepherd	0 (0/3)	66.67 (2/3)	0 (0/3)	0 (0/3)
Pinscher	0 (0/7)	42.86 (3/7)	14.29 (1/7)	0 (0/7)
Pitbull	0 (0/10)	40 (4/10)	20 (2/10)	0 (0/10)
Pomeranian	0 (0/5)	20 (1/5)	0 (0/5)	0 (0/5)
Pug	0 (0/9)	44.44 (4/9)	0 (0/9)	0 (0/9)
Rottweiler	0 (0/1)	100 (1/1)	0 (0/1)	0 (0/1)
Standard Schnauzer	4.76 (1/21)	42.86 (9/21)	9.52 (2/21)	4.76 (1/21)
Sharpei	0 (0/1)	100 (1/1)	0 (0/1)	0 (0/1)
Shih Tzu	0% (0/9)	55.56 (5/9)	11.11 (1/9)	0 (0/9)
Siberian Husky	11.11 (1/9)	44.44 (4/9)	11.11 (1/9)	0 (0/9)
Newfoundland	0 (0/1)	100 (1/1)	0 (0/1)	0 (0/1)
Weimaraner	0 (0/2)	0 (0/2)	0 (0/2)	0 (0/2)
Yorkshire Terrier	5.88 (1/17)	52.94 (9/17)	5.88 (1/17)	0 (0/17)
Region				
Barranquilla	10.42 (35/336)	59.82 (201/336)	20.83 (70/336)	0.59 (2/336)
Puerto Colombia	27.77 (5/18)	100 (18/18)	44.44 (8/18)	0 (0/18)
Total	11.30 (40/354)	61.86 (219/354)	22.03 (78/354)	0.56 (2/354)

From each animal included in the study, 1 ml of blood was collected from the cephalic or jugular vein, from which serum was obtained and stored at −20°C until tested for *D. immitis* antigens and *Anaplasma* spp., Lyme disease and *Ehrlichia* spp. antibodies by the rapid‐assay SNAP 4DX PLUS (IDEXX Laboratories, Inc., Westbrook, Maine, USA) following the manufacturer's instructions. The sensitivity and specificity, respectively, reported by the manufacturer, were 99.0% and 99.3% for *D. immitis* using necropsy, 90.3% and 94.3% for *Anaplasma* spp. using IFA, 97.1% and 95.3% for *Ehrlichia* spp. using IFA (*E. canis*) and ELISA (*E. ewingii*) and 94.1% and 96.2% for *Borrelia burgdorferi* using IFA.

### 2.3 Geographic information system

A map of the sampling area was constructed using QGIS 3.10.1 software and Google maps including all layers of relevant environmental information (rivers, irrigated croplands and parks). All dog samples were georeferenced by GPS at the point of capture. The map shows georeferenced positive dogs by diseases.

### 2.4 Statistical analysis

The data were analyzed using the SPSS Base 18.0 software. A descriptive analysis of the variables was carried out and a chi‐squared test was performed to compare proportions for the qualitative variables, to an *α* = 0.05 significance level.

## 3 RESULTS

The highest seroprevalence was 61.86% (219/354) for *Ehrlichia* spp., followed by 22.03% for *Anaplasma* spp. (78/354) and 0.56% (2/354) for *B. burgdorferi* with the prevalence of *D. immitis* being 11.30% (40/354). In addition, some dogs positive for antibodies against two or more infectious diseases were found: 68 (19.29%) were positive for *D. immitis* + *Ehrlichia* spp. + *Anaplasma* spp., 54 (15.25%) for *Erlichia* spp. + *Anaplasma* spp. and 19 (5.37%) for *D. immitis* + *Ehrlichia* spp. The results by age, sex, habitat and location are shown in Table 1. When evaluating data by age, significant differences were found between 3 and 4.5 and < 1 year‐old groups in *D. immitis* (*p* = 0.0213), 4.6 and 7.4 and < 1‐year‐old groups in *Ehrlichia* spp. (*p* = 0.0112), and between the 6–7.4‐year‐old group and all other age groups (*p* = 0.0310) in *B. burgdorferi*. Female dogs had higher seroprevalences for *Ehrlichia* spp. and *Anaplasma* spp., and higher prevalence of *D. immitis*, compared to male dogs, but this difference was not significant. Dogs that lived outdoors had significantly higher seroprevalence of *Ehrlichia* spp. and *Anaplasma* spp. and higher prevalence of D. *immitis* compared to dogs living indoors (*p* = 0.0019). By breed, only Beagle and Standard Schnauzer tested positive for all canine vector‐borne diseases studied. The rest of the breeds tested positive for one or three infectious diseases. Of the 354 dogs in the study, 325 dogs were clinically healthy, while clinical signs were recorded in 29 dogs (8.2%) 1.6% of cases suffered from cough after exertion and shortness of breath, lack of appetite and weight loss, and were infected with *D. immitis*. The remaining clinical cases were seropositive for *Ehrlichia* spp. (72.1%) and *Anaplasma* spp. (26.3%) and showed lethargy, anorexia, anaemia, digestive disorders, alopecia as well as dermatitis, ulcerative lesions and onychogryphosis. No patients seropositive for *B. burgdorferi* exhibited any clinical signs.

Samples of positive dogs were georeferenced on a map where layers of relevant geographic and environmental information were included (Figure 1). All positive cases were located in the vicinity of some of rivers, areas with stagnant water, parks, mountainous areas or even in coastal areas.

## 4 DISCUSSION

The objective of this study was to analyze and monitor the current epidemiology of four high‐impact vector‐borne diseases in domestic dogs in the area of Barranquilla and Puerto Colombia, Atlántico, Colombia.

In the study population, *Ehrlichia* spp. had the highest seroprevalence, followed by *Anaplasma* spp., *D. immitis* antigen and *B. burgdorferi*. Moreover, high percentage of dogs with antibodies against *D. immitis*/*Ehrlichia* spp./*Anaplasma* spp.; or *Ehrlichia* spp./*Anaplasma* spp.; or *D. immitis* spp./*Anaplasma* spp. were found. A study carried out in the same area (L. Labarthe et al., 2018) reported much lower prevalences or seroprevalence rates than those obtained in the current study, both in infections and co‐infections, even though the sample sizes were very similar in both studies, taking into account that most of the dogs analyzed in this study are outdoor. In addition, there are nearby regions with climates similar to Colombia's, where infection rates are lower, and where there is no rigorous monitoring, which could lead to a rise in the number of infected animals if appropriate control measures are not taken. These differences should be interpreted with caution, as methods of sample collection and/or sensitivity and specificity of the diagnostic tests in each study vary (Esteban‐Mendoza et al., 2020; N. Labarthe & Guerrero, 2005; McCown et al., 2014; Miranda et al., 2009; Pesapane et al., 2019; Vargas‐Hernández et al., 2016). For this reason, it would be interesting to carry out a large‐scale pathogen‐detection survey using the same diagnostic methods, evaluating for standardized set of clinical signs and risk factors in order to unify criteria and compare results more effectively.

Humidity and temperature are fundamental factors for the establishment of parasitic diseases (Otranto et al., 2017). In Barranquilla and Puerto Colombia, according to the Institute of Hydrology, Meteorology and Environmental Studies of Colombia (2021), the average annual humidity is 85%, and at many times of the year it reaches 100%. Different species of Culicidae mosquitoes and ticks have been reported in several locations throughout the national territory and close to the study area (L. Labarthe et al., 2018; N. Labarthe & Guerrero, 2005; Miranda & Máttar, 2015; Montoya‐Lerma et al., 2011; Quintero Espinosa, 2015). In this study, there were a high number of positive dogs that lived outdoors and close to or within green areas, near river banks or river mouths, both inside and outside urban areas. Moreover, if we take into account that there is abundant vegetation and water present (river banks, parks and irrigated croplands) and high humidity, factors that promote the proliferation of mosquito vectors, in those areas where positive dogs have been detected, the risk of CVBD infection and its effect on public and animal health is likely to be significant throughout the year. While there are no studies on the incidence of these zoonoses in our study area, prior literature studying areas with similar environmental characteristics has documented that the presence of animals infected with zoonotic CVBDs coincides with the detection of human infections in the area (L. Labarthe et al., 2018; Maggi & Krämer, 2019; Rodríguez‐Morales et al., 2019; Tsachev et al., 2006).

The prevalence or seroprevalence of CVBDs shown in this study is quite high, suggesting that control and prophylaxis programmes should be implemented on dog populations and mosquito and tick populations in the study area. These programmes should be implemented by veterinarians or personnel qualified in the study of these vectors where most dogs live outdoors. Veterinarians should be made aware of the importance of these diseases, in order to implement appropriate control campaigns and raise awareness among pet owners. In addition, further studies addressing the geolocation of infected or seropositive dogs are needed to understand the full extent of the problem and take the most efficient control measures for both animal and human populations.

## 5 CONTRIBUTION TO THE FIELD STATEMENT

CVBDs have zoonotic potential and pose a potential risk to public health. In Colombia, there are very few studies that address the prevalence of these diseases. The objective of this study was to update the prevalence of cardiopulmonary dirofilariosis and the seroprevalence of anaplasmosis, ehrlichiosis and Lyme borreliosis in dogs in Barranquilla, northern Colombia. The present study included 354 dogs presented to veterinary clinics for routine health examination using the commercially available SNAP 4DX PLUS Test. The seroprevalence of dogs for *Ehrlichia* spp. was 61.86% (219/354), followed by 22.03% of dogs for *Anaplasma* spp. (78/354), 11.30% (40/354) for *D. immitis* antigens and 0.56% (2/354) for antibodies against *B. burgdorferi*. In addition, we found several positive dogs for antibodies against two or more infectious diseases (*D. immitis*/*Ehrlichia* spp./*Anaplasma* spp., *Ehrlichia* spp./*Anaplasma* spp. and *D. immitis*/*Anaplasma* spp.) with percentages between 5.37% and 19.20%. The highest seroprevalence was found in dogs housed outdoors. The high seroprevalence of *Ehrlichia* spp. and *Anaplasma* spp. found in this study suggests that veterinarians should routinely implement prophylactic programmes for these CVBDs, particularly for dogs that reside outdoors.

## CONFLICT OF INTEREST

Dr. Couto is a consultant for IDEXX Laboratories. The other authors declare no conflict of interest.

## ETHICS STATEMENT

The authors confirm that the ethical policies of the journal, as noted on the journal's author guidelines page, have been adhered to and the appropriate ethical review committee approval has been received. Helsinski Declaration of the code of ethics and animal welfare for the Care and Use of Animals were followed.

## AUTHOR CONTRIBUTIONS

Óscar Otalora and Rodrigo Morchón designed the study and wrote the manuscript. Guillermo Couto, Julio Benavides and Carlos Mucha performed the fieldwork and collected the data and performed the experiments. All authors participated in the discussion of the results, as well as in correcting and approving the final manuscript.

### PEER REVIEW

The peer review history for this article is available at https://publons.com/publon/10.1002/vms3.673

## Data Availability

The data that support the findings of this study are available from the corresponding author upon reasonable request.
